# Morphological and Molecular Characterization of Cyanobacteria Isolated from Two Geothermal Springs of the Central Ecuadorian Andes

**DOI:** 10.3390/microorganisms13122763

**Published:** 2025-12-04

**Authors:** Renato E. Naranjo, Andrés Izquierdo

**Affiliations:** 1 Departamento de Ciencias de la Vida y la Agricultura, Universidad de las Fuerzas Armadas ESPE, Sangolquí 171103, Ecuador; renatonaranjo53@gmail.com; 2 Grupo de Investigación en Microbiología y Ambiente GIMA, Universidad de las Fuerzas Armadas ESPE, Sangolquí 171103, Ecuador; 3 Centro de Nanociencia y Nanotecnología CENCINAT, Universidad de las Fuerzas Armadas ESPE, Sangolquí 171103, Ecuador

**Keywords:** cyanobacteria, geothermal spring, morphology, molecular phylogeny

## Abstract

Geothermal springs are scattered worldwide and harbor thermophilic cyanobacteria, whose species distribution depends on extreme environmental conditions. The optical growth of cyanobacteria in hot springs and their metabolic diversity represent a source for possible biotechnological tools. In the current study, we isolated and characterized the cyanobacterial community of water samples from El Salado and Papallacta geothermal springs in Ecuador. We employed a culture-dependent as well as a polyphasic approach, which includes morphological examination by light microscopy, molecular analysis of *16S* and *23S* rRNA genes, and phylogenetic position using the maximum likelihood method. Notably, the morphological and molecular analyses complemented each other. Furthermore, we isolated eleven strains that belong to the taxa *Tenebriella amphibia*, *Calothrix*, *Planktothricoides* raciborskii, *Nostoc*, *Fischerella muscicola*, *Leptolyngbya*, *Synechococcus*, *Komvophoron jovis*, Chroococcales and Nostocacea. Both hot springs, whose temperature ranged between 45 and 54 °C, could harbor cosmopolitan and endemic cyanobacteria. Our study establishes a baseline for future efforts to exploit potential biotechnological tools bioprospected from these isolated microorganisms.

## 1. Introduction

Cyanobacteria constitute a phylum of crucial importance in ecology and evolution. They provide the basis for ecosystem stability through photosynthesis, atmospheric nitrogen fixation, and phosphorus accumulation [1,2,3]. These autotrophic prokaryotes present a broad range of cellular strategies, physiological capacities, and adaptations including the significant ability to thrive in diverse habitats such as freshwaters, the marine environment, and extreme habitats such as geothermal springs commonly known as “hot springs” [4,5,6,7]. Geothermal springs are scattered worldwide, primarily associated with current o recent volcanic activity [4]. Moreover, their hot waters may harbor endemic species of thermophilic cyanobacteria due to geographic isolation and possible evolutionary divergence [8]. The distribution pattern of these species varies with temperature and pH, in combination with availability of combined nitrogen, phosphorus and/or concentration of free sulfide within extreme environmental conditions [4].

*Thermosynechococcus elongatus*, *Chlorogloeopsis*, *Leptolyngbya*, *Fischerella*, *Geitlerinema*, *Pleurocapsa,* and *Calothrix* well-known cyanobacteria capable of tolerating the highest temperatures [5,9,10,11]. Representative cyanobacterial strains of geothermal habitats have been studied in numerous regions of the word such as Algeria [1], India [10,12], Sri Lanka [13], Iran [14], China [2], Taiwan [15], Greece [16], France [7], Bulgaria [17], Italy [18], and Northern Iceland [5]. Most thermophilic cyanobacteria are metabolically diverse and possess stable enzymes under extreme environmental conditions [19]. For instance, polyphosphate kinase from *T. elongatus* was successfully employed in an ATP regeneration system that could be used at high temperatures to effectively produce dipeptides [20]. Hence, isolation, axenic cultivation, and taxonomic characterization of strains yield a source of organisms for possible biotechnological exploration [10,12]. Patel et al. [11] reviewed some applications of thermophilic cyanobacteria such as CO_2_ emotions reduction to generate high added-value bioproducts, biofuel production, waste bioremediation, production of economically viable pigments and bioactive compounds, and the development of microbial fuel cells.

Morphological characterization provides an overall evaluation of cyanobacterial diversity and abundance of microbial communities in different environments [13]. However, cyanobacteria classification based solely on morphological criteria can be challenging, as morphological features often vary depending on environmental conditions, and cryptic species may exhibit similar morphologies but are genetically distinct. One of the most prominent examples is *Phormidium* [5,10,21,22]. Hence, identification under optical microscopy is generally limited to species with simple morphological features, such as the tiny coccoid cyanobacteria or the very thin filamentous Oscillatoriales [17]. Therefore, a polyphasic approach incorporating morphological and molecular data is recommended to correctly identify species [21,23]. The *16S* rRNA gene is the most used marker in cyanobacterial systematics located in different environments at a global and regional scale, contributing to the analysis of distribution and evolution [4,18]. On the other hand, Domain V of the *23S* rRNA gene is a plastid marker for multiple groups of eukaryotic algae and cyanobacteria [24].

The Ecuadorian Quaternary volcanic arc is comprised by at least 250 volcanoes [25]. Some of these volcanoes are associated with hydrothermal systems [26]. The National Institute of Meteorology and Hydrology of Ecuador has identified 119 hot springs, predominantly located along the Andean region and the southern Coast. These hot springs exhibit average temperatures of approximately 31.3 °C, with maximum temperatures reaching up to 74 °C [27,28]. Most of these hot springs are of significant socio-economic importance due to their recreational, therapeutic, industrial, and religious uses [27]. A few studies have revealed the diversity of cyanobacteria communities in Ecuadorian geothermal springs. *Phormidium* sp. and *Leptolyngbya* sp. were reported in the hot springs of San Vicente and Aguas Hediondas [29]. Subsequently, Rivas et al. [30] isolated a cyanobacterium strain closely related to *Chroococcidiopsis thermalis* from Aguas Hediondas at temperatures around 54 °C and a pH of 4.8.

In the present study, the cyanobacterial communities of El Salado and Papallacta geothermal springs were isolated and characterized using a combination of classical morphological examination and molecular analyses of the *16S* and *23S* rRNA genes. As this is the first report to describe cyanobacterial diversity from thermal environments in the inter-Andean region of Ecuador, we expect that our study will serve to encounter further potential strains for biotechnological applications. This publication is the result of a 10-year-old dataset that had never been written up for peer-review and dissemination. Strain insolation, morphological characterization, sequencing and the initial bioinformatic analysis were performed in 2015–2016. To enhance the robustness and relevance of our findings, the bioinformatic analysis were updated in 2023.

## 2. Methods

### 2.1. Sample Collection

**Figure 1:**
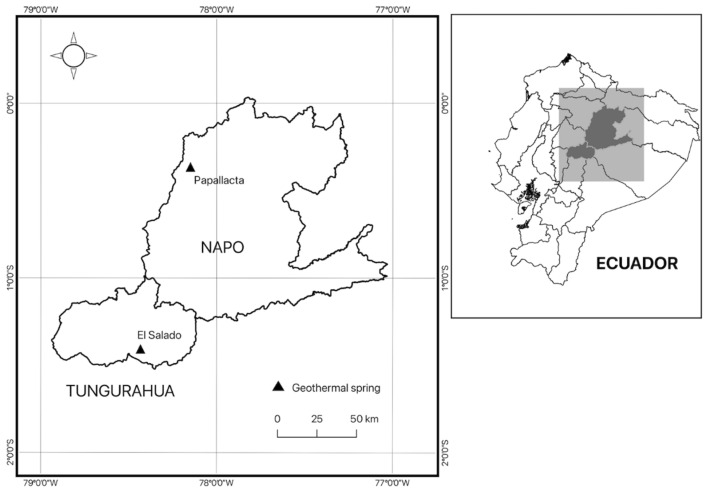
Locations of two invested geothermal springs of Andean region.

**Figure 2:**
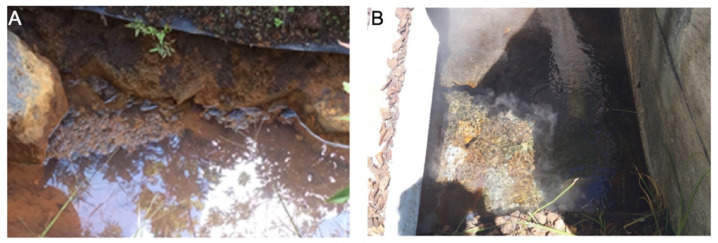
Sampling sites: (**A**) El Salado spring; (**B**) Papallacta spring.

Water samples with sediment from two geothermal springs were collected from September through October in 2015. The sampling sites in Ecuador were El Salado (S 1°24′17.973″, O 78°25′58.5″), at 1903 m.a.s.l., close to Río Negro, in the Province of Tungurahua, and Papallacta (S 0°21′45.284″, W 78°8′57.453″) at 3300 m.a.s.l., close to Quijos, in the Province of Napo (Figure 1). The two sampling sites are depicted in Figure 2. El Salado spring was located within a channel being roughly 1.5 m wide, whose flow forms small basins downstream, while the Papallacta spring was enclosed by concrete walls with slight access to light sources. 500 mL sterile glass bottles were affixed to a metal clamp with an extended handle. Each bottle was immersed at the sampling site until it was filled with water, after which it was sealed and labeled. These bottles were transported in a polystyrene box at room temperature to the Environmental Microbiology laboratory at the Centro de Nanociencia y Nanotecnología (CENCINAT) of the Universidad de las Fuerzas Armadas ESPE for sample processing.

### 2.2. Physicochemical Analysis

The temperature and pH of the water were measured at each sampling point using a portable digital thermometer and a pH meter (PmoYoko, Jinan, China). Then, we collected 3 L of water in sterile plastic bottles, which were then transported in a polystyrene box with ice. Physicochemical properties analyses of these water samples were outsourced to two accredited laboratories in Ecuador (CESAQ-PUCE and Labanncy Ltd., Quito, Ecuador).

### 2.3. Isolation of Cyanobacteria and Morphological Identification

A 100 µL aliquot of sample was inoculated using the spread plate method onto Petri dishes containing sterile BG-11 (for non–nitrogen-fixing strains) or BG-11o (for nitrogen-fixing strains) medium solidified with 1.5% (*w*/*v*) agar. Plates were incubated at 20 ± 2 °C under a 12 h light/12 h dark photoperiod using fluorescent illumination (average 16 μmol photons m^−2^ s^−1^) for 15 days. Colonies were examined under a stereomicroscope (Olympus, Japan), and isolated colonies were transferred to fresh medium. This procedure was repeated five times to ensure purification. Additionally, unicellular cyanobacteria lacking filaments were isolated using a short-tip Pasteur pipette or capillary. A droplet of the sample was placed on a slide and observed under a light microscope (Olympus, Tokyo, Japan). Individual cells of interest were drawn into the pipette by capillary action and transferred into a drop of sterile medium on a clean slide. This process was repeated until only a single cell type remained in the drop. Purified strains from both solid and liquid cultures were expanded in 10 mL of the corresponding medium for DNA extraction.

Cyanobacteria (referred to as “Cyanoprokaryota” in Komárek and Anagnostidis’ works) were identified under an optical microscope and a digital microscope (Luminoptic, Melbourne, VIC, Australia) with a camera of 5.0 megapixels using the taxonomic information of Komárek et al. [31], Komárek and Anagnostidis [32], Komárek [33], Anagnostidis and Komárek [34], Anagnostidis [35], and Algaebase [36]. Each cyanobacterium was described through micrographs, and the measurements of vegetative cells and heterocysts were obtained using the ScopeImage-software version 9.0 and Fiji-software version 1.53t.

### 2.4. Molecular Analysis of Cyanobacterial Isolates

Genomic DNA was isolated from all cyanobacteria strains using the method described by Cai and Wolk [37] with some modifications. Briefly, 2 mL of cyanobacteria samples was transferred to 2 mL centrifuge tubes, and centrifuged for 5 min at 14,000 rpm. After supernatant was discarded, three sterile 2 mm glass beads, 500 μL of TE *buffer* (10 mM Tris-HCl, 0.1 mM EDTA, pH 7.5), 70 μL of 10% sodium dodecyl sulfate (Sigma-Aldrich, Burlington, MA, USA), and 225 μL of phenol-chloroform (1:1, *v*/*v*) were added to the tubes. Samples were vigorously vortexed for 1 min followed by cooling on ice for 1 min. This process was repeated for a total of five times. The suspensions were centrifuged for 15 min at 14,000 rpm, while the supernatants from each extraction were transferred to new tubes. Then, two phenol extractions were performed. For each extraction, 700 uL of phenol pH 8.0 were added, samples were vortexed and centrifuged for 2 min at 14,000 rpm, and the supernatants were placed in separate tubes. DNA was precipitated using 150 μL of 3 M sodium acetate (pH 5.2) and 300 μL absolute ethanol. The tubes were stored at −20 °C for 3 h, centrifugated for 10 min at 14,000 rpm, while the supernatant was discarded. DNA pellets were washed twice with 70% ethanol and resuspended in 75 μL of nuclease-free water (Santa Cruz, CA, USA), and two units of RNase A (Thermo Fisher Scientific Inc., Waltham, MA, USA) were added. Suspensions were incubated at 37 °C for 15 min at 400 rpm, and DNA samples were stored at −20 °C until further use.

The *16S* and *23S* rRNA genes were amplified using specific primers (CYA106F: 5′-CGGACG GGTGAGTAACGCGTGA-3′, CYA781R: 5′-GACTACTGGGGTATCTAATCCCATT-3′) [38], and (p23SrV_f1: 5′-GGACAGAAAGACCCTATGAA-3′, p23SrV_r1: 5′-TCAGCCTGTTATCCCTAGAG-3′) [24]. PCR reactions were conducted in 25 μL, and the amplification solution contained 12.5 μL 2X GoTaq Green Master Mix (Promega, Madison, WI, USA), 1.25 μL of each primer (10 μM), 7.5 μL nuclease-free water, and 2.5 μL of DNA. Conditions for the *16S* gene amplification were initial denaturation at 94 °C for 2 min followed by 35 cycles at 94 °C for 20 s, 55 °C for 30 s, and 72 °C for 30 s, and final extension at 72 °C for 10 min. On the other hand, conditions for the *23S* gen amplification were initial denaturation at 95 °C for 3 min, 35 cycles at 94 °C for 30 s, 60 °C for 30 s, and 72 °C for 1 min, and final extension at 72 °C for 10 min. PCR products were visualized by running a 1% agarose gel stained with Gel Star (Lonza, Rockland, ME, USA). Amplicons were bidirectionally sequenced using the Sanger method (Macrogen, Seoul, Republic of Korea). The obtained sequences were analyzed with Geneious R9.1 [39] to obtain consensus sequences. BLASTn 2.14.0 searches [40] were used to initially infer sequence identity by pairwise comparisons with homologs in GenBank database. Sequences generated in this study were deposited in the GenBank database under the accession numbers MH090926-MH090932 (*16S* rRNA gene) and MH101455-MH101461 (*23S* rRNA gene).

### 2.5. Phylogenetic Analysis

To investigate evolutionary relationships, homologous *16S* and *23S* rRNA gene sequences from cyanobacterial species were retrieved from the GenBank database (as of July 2023), selected based on BLAST similarity to our study strains. Retrieved sequences were screened to exclude incomplete or low-quality entries, and only those covering at least 90% of the full gene length were retained for analysis. To ensure broad taxonomic representation, sequences were selected to maximize diversity across the orders Nostocales and Oscillatoriales, and appropriate outgroup taxa were included.

Multiple sequence alignments were performed separately for the *16S* and *23S* rRNA datasets using the MUSCLE algorithm [41] implemented in MEGA version 11.0.13 [42], with default parameters. Ambiguous regions and gaps were removed using Gblocks [43], with default parameters, to retain phylogenetically informative sites.

Phylogenetic trees were constructed with the maximum likelihood (ML) method in MEGA, using the Kimura 2-parameter substitution model with gamma distribution and invariant sites (K2 + G + I) for both genes. The best-fitting evolution model for each alignment was determined based on the Bayesian Information Criterion (BIC) using the “Find Best DNA/Protein Models (ML)” tool. Node support was assessed with 1000 bootstrap replicates per branch. Trees were visualized and edited using Tree explorer tool.

## 3. Results

### 3.1. Physicochemical Analysis of Hot Spring Waters

The measured physicochemical parameters are listed in Table 1. In situ analysis revealed that the hot water from the Papallacta spring had higher temperatures than the El Salado spring. Additionally, El Salado has a slightly acid pH range, while Papallacta has a neutral to nearly alkaline pH. Laboratory analysis indicated high conductivity in the two water springs, demonstrating a higher value in Papallacta. El Salado spring exhibited high concentrations of Cl^−^, SO_4_^−2^, K^+^, Ca^2+^, and Mg^2+^; whereas Papallacta spring had considerable Cl^−^, Na^+^, and Ca^2+^ concentrations. Moreover, the alkalinity of El Salado’s water was significantly higher than of Papallacta’s water. The values of arsenic and copper were almost identical in the two sampling points, while El Salado’s water contained a high concentration of iron.

### 3.2. Isolation and Morphological Identification

**Figure 3:**
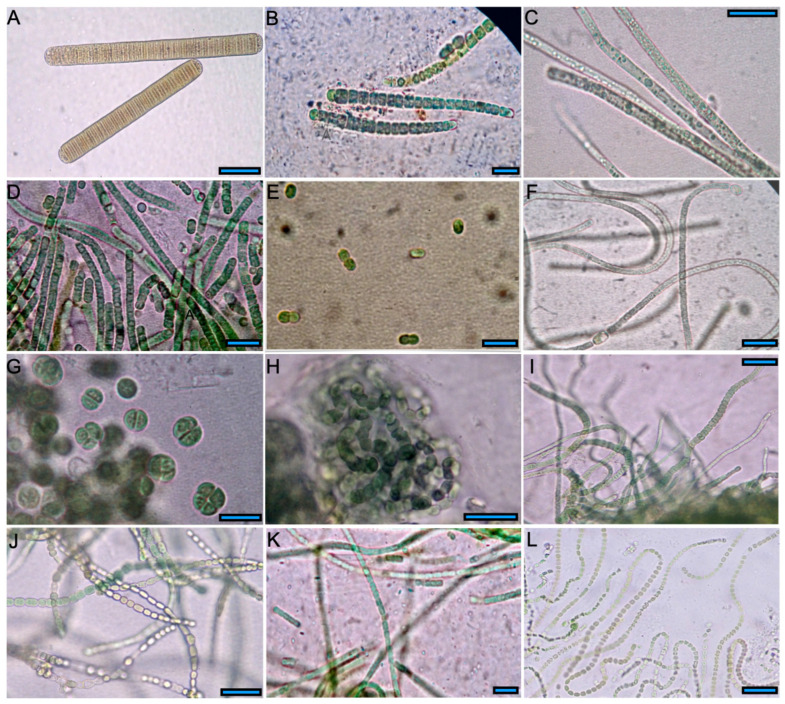
Images of cyanobacteria isolated in BG-11 medium and identified under the optical microscope. (**A**) *Tenebriella amphibia* B2A; (**B**) *Calothrix* sp. B3A; (**C**) *Planktothricoides* sp. B4A; (**D**) *Leptolyngbya* sp. B5A; (**E**) *Synechococcus* sp. B6A; (**F**) *Nostoc* sp. P11A; (**G**) Chroococcales P12A; (**H**) Nostocales P13A; (**I**,**J**) *Fisherella* sp. P14A; (**K**) *Leptolyngbya* sp. P15A; (**L**) *Komvophoron jovis* P38A. Scale bar: 30 µm in (**A**), scale bar: 20 µm (**C**,**F**–**J**,**L**), scale bar: 10 µm in (**B**,**D**,**E**,**K**).

In the present study, eleven strains were isolated and identified as belonging to the orders Nostocales (4), Oscillatoriales (3), Synechococcales (3), and Chroococcales (1). Only the genus *Leptolyngbya* was isolated from the two springs. Cyanobacterial morphotypes are depicted in Figure 3, and Table 2 lists the measurements of vegetative cells and heterocysts.

B2A was identified as *Tenebriella amphibia*, with brownish cells being wider than long, formed solitary and extended trichomes with rounded apical cells (Figure 3A). B3A (*Calothrix* sp.), exhibited short filaments with thin sheaths comprising conical blue-green cells. The number of vegetative cells was up to 20, and their size decreased toward the basal part of the trichome. This morphotype also indicated a solitary spherical apical heterocyst (Figure 3B). B4A (*Planktothricoides* sp.) consisted of long tangled trichomes with rounded apical cells and gas vacuoles (Figure 3C). Its large colonies penetrated the solid medium. B5A and P15A (*Leptolyngbya* sp.), presented constricted trichomes in dense clusters, composed of green vegetative cells slightly longer than wide and an inconspicuous sheath (Figure 3D,K).

B6A (*Synechococcus* sp.) consisted of blue-green solitary cells with a spherical and elongated shape before division (Figure 3E). P11A (*Nostoc* sp.) indicated brownish cylindrical vegetative cells forming long trichomes, while spherical heterocysts were present in the terminal or intermediate position (Figure 3F). P12A was identified as species of the Chroococcales order with solitary or agglomerated blue-green cells. These cells were spherical or slightly oval before division, without mucilaginous envelopes (Figure 3G). P13A (Nostocales order) presented green oval cells formed short coiled trichomes, grouped and embedded in a mucilaginous matrix. Spherical heterocysts less broad than vegetative cells were also present (Figure 3H). P14A (*Fischerella* sp.) presented primary filaments and true-branched filaments composed for barrel-shaped green cells. We observed brownish-colored cylindrical heterocysts in the branches. Furthermore, the colonies penetrated the agar and displayed a distinctive earthy/musty smell, typical of geosmin [44] (Figure 3I,J). P38A (*Komvophoron jovis*) consisted of brownish cells with hemispherical and conical shapes that formed large twisting trichomes without branches. A hardly distinguishable hyaline sheath rounded the small vegetative cells, which were slightly separated (Figure 3L).

### 3.3. 16S and 23S rRNA Gen Phylogenetic Analysis

Analysis of *16S* and *23S* rRNA partial sequences was performed for seven strains. BLAST search of *16S* rRNA sequences of B2A, B4A, P11A and P14A yielded > 99% similarity to other cyanobacteria sequences in the GenBank database (Table 3). Except for P12A, the remaining strains reported between 97 and 98% similarity. Likewise, the *23S* rRNA sequence of P14A determined >99% homology. However, the other sequences reported homologies <97%.

**Figure 4:**
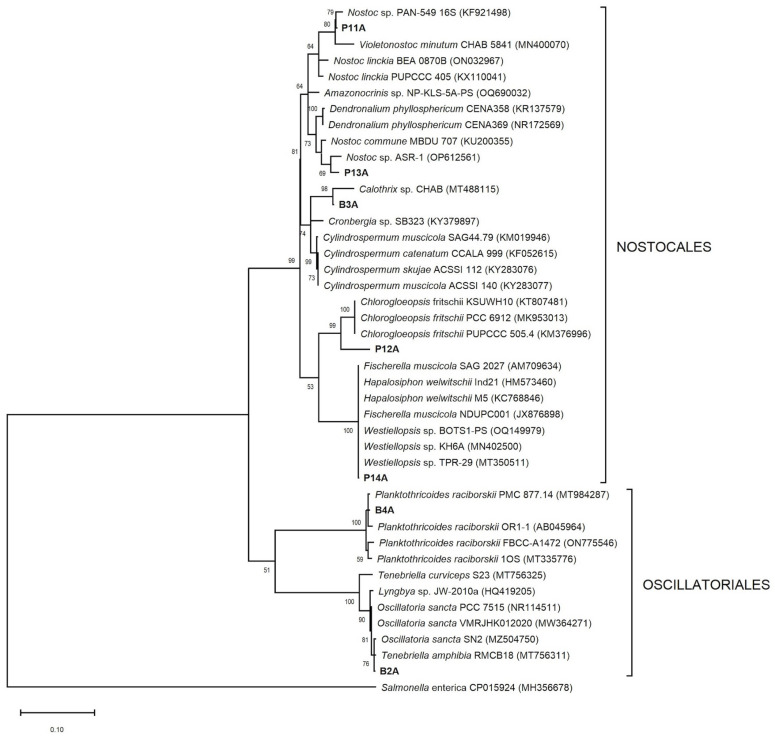
Maximum likelihood phylogenetic tree based on partial *16S* rRNA gene sequences of representative cyanobacteria belonging to the order Nostocales and Oscillatoriales. Sequences obtained in the present study are indicated in bold, while other sequences were obtained from the GenBank. Numbers near nodes indicate bootstrap values above 50% from 1000 replicates. *Salmonella enterica* CP015924 was used as an out-group. GenBank accession numbers are shown in parentheses. Scale bar is of 10% substitutions per site.

**Figure 5:**
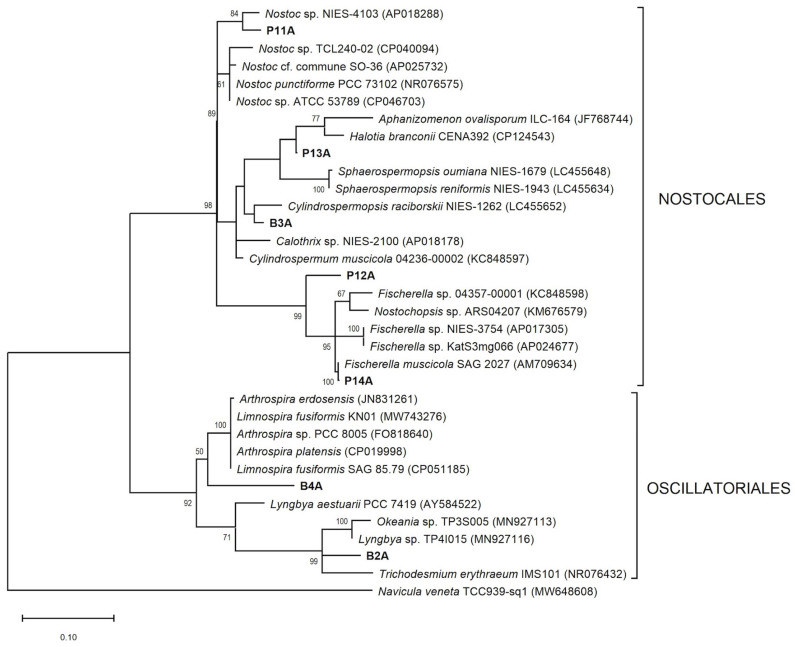
Maximum likelihood phylogenetic tree based on partial *23S* rRNA gene sequences of representative cyanobacteria belonging to the order Nostocales and Oscillatoriales. Sequences obtained in the present study are indicated in bold, while other sequences were obtained from the GenBank. Numbers near nodes indicate bootstrap values above 50% from 1000 replicates. *Navicula veneta* TCC939-sq1 was used as an out-group. GenBank accession numbers are shown in parentheses. Scale bar is of 10% substitutions per site.

The phylogenetic analysis demonstrated that non-heterocystous strains (Oscillatoriales) were organized in a different cluster from heterocystous strains (Nostocales) into the two trees (Figure 4 and Figure 5). Moreover, B2A and B4A strains clustered with Oscillatoriales cyanobacteria but were in distinct internal clades. On the *16S* rRNA phylogenetic tree, B2A formed a clade with another *T. amphibia* and Oscillatoria sancta sequences, while B4A and *Planktothricoides raciborskii* sequences formed a clade with a strong bootstrap value (100%). B3A formed a clade with *Cilindrospermum* species and a subclade with another *Calothrix* strain. P11A and P13A, for their part, were grouped in a *Nostoc* clade, each forming a subclade with unpublished sequences of *Nostoc* sp. P12A and P14A were a distinct clade from the previously described heterocystous strains. Although P12A was identified as Chroococcalean cyanobacterium under the microscope, the phylogenic analysis indicated a strong clustering (99% bootstrap) with *Chlorogloeopsis fritschii* sequences. P14A clustered strongly (100% bootstrap) with *Fisherella muscicola* SAG2027 and unpublished sequences of *Fisherella, Westiellopsis*, and *Hapalosiphon*. *Westiellopsis* sp. TPR-29 was reported from an Indian thermal spring. *23S* rRNA phylogenetic tree is depicted in Figure 5, where P3A formed a clade with *Calothrix* sp. NIES-2100 and P13A strain. Unlike *16S* rRNA tree, P13A was outside the *Nostoc* clade and grouped in a clade with *Halotia branconni*. P12A and sequences of Hapalosiphonaceae family formed a highly supported group (99%) in which P14A and two *Fisherella* sequences (AP017305, AP024677) from Japanese hot springs were present. Finally, like *16S* rRNA tree, the insolate P11A clustered with members of the *Nostoc* genus.

## 4. Discussion

The current research supported the importance of morphological characterization and molecular analysis to study the diversity of cyanobacteria in hot springs of the Andean mountain. By culture-dependent approach, we isolated cyanobacteria representatives of the genera *Tenebriella*, *Planktothricoides*, *Komvophoron*, *Calothrix*, *Nostoc*, *Fischerella*, *Leptolyngbya*, *Synechococcus*, and two unidentified genera of Chroococcales and Nostocacea, from hot water from El Salado and Papallacta geothermal springs. Gene sequences and phylogenetic analysis allowed to corroborate the morphological identification and increase the resolution of taxonomy to classify our strains. Both hot springs evidenced diverse communities of cyanobacteria. Papallacta spring yielded the highest temperature (50–54 °C), where heterocystous filamentous forms (Nostocales) were predominated. The presence of heterocysts relies on the availability of combined nitrogen at temperatures below ~55 °C [4]. However, in some springs with high temperatures (~63 °C), combined nitrogen may be deficient and heterocystous cyanobacteria may thrive [1,4].

The P14A strain is the most closely related to *F. muscicola,* with the highest percentages of identity in the analysis of both genes (Table 3). *Fischerella* is a frequent constituent of microbial communities in hot springs, typically found up to 58 °C [1,12,45]. Its adaptation to high temperatures could be due to a high abundance of phycobilisome linkers or faster turnover of photosystem proteins that this genus presents [46]. According to Kaštovský and Johansen [47], some strains of *Fischerella* are misidentified because the authors frequently identified *Mastigocladus* (reserved for populations with true branching from thermal springs) as *Fischerella*. This explains the presence of *F. major* and *F. muscicola* in the thermal springs clade despite being from terrestrial or aerial habitats. Therefore, P14A could possibly be identified as *Mastigocladus*. Nevertheless, we maintain it as *F. muscicola*. Only P14A strain was closely related to sequences of geothermal microorganisms. In the *23S* rRNA analysis, this strain matched with *Fischerella* sp. strain NIES-3754 [48] and uncultivated *Fischerella* sp. KatS3mg066 [49] with 97% similarity, both from hot springs in Japan. However, the closest match did not derive from a hot spring (*Fischerella muscicola* SAG2027, with a 99% similarity). On the other hand, P14A strain was 100% similar to species of genera *Hapalosiphon, Fischerella*, and *Westiellopsis*, forming a strong clade with them (Figure 4). The most problematic clusters within Nostocales are genera *Hapalosiphon, Westiellopsis*, *Fischerella, Pelatocladus*, *Nostochopsis*, and *Mastigocladus* [50]. Phylogenetic analysis has shown that these genera are closely related and easily confused morphologically, and form a monophyletic lineage corresponding to one family, being Hapalosiphonaceae [31]. Unfortunately, many of these taxa have been described from very restricted habitats, hence, the true genetic and ecological diversity remains unknown [50]. Other authors recommended an alternative solution to collapse these four genera into a single *Fischerella* genus [51]. In addition, the high match of P14A with species of Hapalosiphonaceae family may be explained since some thermophilic cyanobacteria, such as *Fischerella*, appear in the cosmopolitan distribution [8].

Under the microscope, P12A demonstrated features of the order Chroococcales, but this morphotype has a basal position from *C. fritschii* clade. Despite *C. fritschii* was the closest match with P12A, it had less than 97% similarity based on *16S* rRNA gene sequencing. The main reason is that no sequences in the NCBI database correspond to this taxon. P12A maintains a short distance to *Fischerella* clade in *16S* and *23* rRNA tree. Strunecký et al. [17] indicated that *Chlorogloeopsis* is phylogenetically very close to the thermophilic *Mastigocladus*/*Fisherella* group. We speculate that the P12A position could reveal the polyphyletic nature of some genera of Chroococcales. Phylogenetic analyses have indicated that many cyanobacteria with simple morphotypes (Chroococcales) are polyphyletic [1,52,53].

Both P11A and P13A strains were identified as species of the family Nostocaceae. The *16S* rRNA tree showed that the P11A and P13A clustered in the *Nostoc* clade with other *Nostoc*-like species (Figure 5). Instead, *23S* rRNA tree demonstrated that these two strains were widely distant, as well as P13A was placed in the clade formed with *Nostoc*-like species, a species of family Calotrichaceae and the B3A strain. *Nostoc* is a polyphyletic group, and the diversity of *Nostoc*-like cyanobacteria is underestimated due to the low genetic and genomic information and the subtle morphological differences often seen among related strains [54]. P11A strain was considered as *Nostoc* sp. because neither morphology nor molecular characterization allowed to give any further species assignment to this organism. Bravakos et al. [16] reported a Nostoc strain isolated from a thermal spring in Greece, whose temperature reached 58 °C. Regarding P13A, the best BLAST for the *16S* and *23S* rRNA sequences were *D. phyllosphericum* CENA369 [54] and *H. branconii* CENA392 [55], respectively. While CENA369 seems to be distantly related to our strain, CENA392 (recently proposed genus *Halotia*) and P13A formed a weak supported clade. Therefore, there is a consensus between the two trees, indicating that P13A could be considered a new taxon of family Nostocacea. We also isolated a heterocystous cyanobacteria (B3A) from El Salado spring. Although B3A had typical *Calothrix* features, phylogenetic analysis only closely related it to clone *Calothrix* sp. CHAB TP201528. In a previous study, *Calothrix* was isolated from hot baths in Iceland, with temperatures between 36 and 40 °C [5]. The metabolic capacities of family Nostocaceae and Calotrichaceae, such as fixing atmospheric carbon and nitrogen and stablishing mutualistic symbioses, could make these families notoriously ubiquitous in different environments [5,10,54].

The rest of the species in this study were from El Salado spring and thrived below 50 °C. Here, the number of non-heterocystous forms (Oscillatoriales and Synechococcales) was higher. Oscillatoriales are considered a major component of the cyanobacterial flora in hot springs worldwide [10,56]. This order is notable in sulfide-rich springs with a temperature range of 40 to 66 °C due to predominance of sulfide-tolerant and sulfide-utilizing species [4,6,57]. Interestedly, the morphological features of family Oscillatoriaceae in cultured isolates and natural populations have been shown to be highly variable, depending on growth and cultivation conditions [36]. Being phenotypic plasticity a taxonomic problem in thin filamentous cyanobacteria [37].

Although the B2A strain was initially identified as *O. sancta*, trichome measurements allowed to recognize this strain as *T. amphibia*. *T. amphibia* differs morphologically from Oscillatoria sancta by trichome width [32,58]. The trichome width of B2A (14.2–19.4 µm) was similar to *T. amphibia* SAG 74.79 (15–19 µm) [58]. Some GenBank sequences clustered within *Tenebriella* were originally named *O. sancta* [58]. This could explain the presence of two species in the B2A clade (Figure 4). Only Bassu et al. [59] have reported the presence of a species of *Tenebriella* in a geothermal environment (Indian spring at 45 °C). On the other hand, *O. sancta* was previously identified in Roman Baths (Italy) a temperature range between 39 and 46 °C [60]. Another Oscillatorialean was B4A. These strains matched with *P. raciborskii* PMC 877.14 (99% similarity), which was isolated from muds of the thermal springs of Balaruc-les-Bains (France) [7]. Molecular phylogeny placed B4A in a main strong clade with other *P.* *raciborskii* sequences (Figure 5). This species was reported in a bathing tank of the Nunbel hot spring (India) at 45 °C [59]. Morphologically, B4A strain indicated narrower cells (2.4–5.9 µm) than cells of PMC 877.14 (5–7.3 µm) [7] and Indian cyanobacteria (4.7–7.6 μm) [59].

In four strains (B5A, B6A, P15A and P38A) from the present study, the polyphasic characterization could not be completed, and only the morphological identification and description were performed. We obtained low-quality sequences, and the possible cause could have developed in the first steps of DNA extraction (cellular lysis). The DNA extraction of *Leptolyngbya* and *Komvophoron* strains could likely be compromised since their sheathes or compact colonies, either of which may interfere with cell disruption [61,62], affecting subsequent DNA purification and gene amplification steps [1]. B5A from El Salado and P15A from Papallacta springs were identified as *Leptolyngbya*, a genus that is often the most abundant in thermal environments [1,18,63]. The B5A and P15A strains composed cells with similar length and width (roughly 2.5 µm) (Table 2). *Leptolyngbya* strains from Euganean Thermal District (Italia) and Geyser Hot Spring (USA) were composed of cells distinctly longer than wide [18]. On the contrary, *Leptolyngbia* cells of thermal muds from France were shorter than wide [7]. Our strains had a similar length to the Italian cyanobacteria. The B6A strain were assigned to genus *Synechococcus*. This species has been isolated in several studies of cyanobacteria flora from thermal habitats [1,2,10,12,13,16,17]. *Leptolyngbya* and *Synechococcus* may be found together below approximately 58 °C [4], and their occurrence in geothermal springs suggests that there is no geographical barrier to the dispersal of these thermophilic taxa [1]. Finally, P38A strain was identified as *K. jovis* by morphological characterization. Our description and measurements of length and width were similar to the information reported by Anagnostidis and Komárek [34]. This species was recently recorded in an Indian geothermal spring at 60 °C [59].

Temperature is not the only the extreme condition for microorganisms in hot springs, but also elevated concentrations (50–150 mg/L) of inorganic ions (Ca^2+^, Mg^2+^, Na^+^, HCO_3_^−^, SO4^2−^, Cl^−^, Si and H_2_S) and elevated or acidic pH [64]. Our findings of possible dominant ionic species are consistent with some previous reports, in which Papallacta is considered a hot spring of the type near-neutral Chloride–Alkali (Na^+^, Ca^2+^) [65], and El Salado spring is Chloride–Sulfate–Alkali (K^+^, Ca^2+^, and Mg^2+^) [66]. The conductivity of water depends on the concentration of dissolved ions and the temperature [10]. Therefore, the high levels of dissolved ions may contribute to the higher measured conductivity in both geothermal springs. Cyanobacteria diversity seems quite limited below pH 6 in hot springs around the world [4], and alkaline pH conditions are more favorable for the growth of many cyanobacteria [13]. Therefore, the pH (6.3 to 7.5) of Ecuadorian springs would be able to promote the growth of the encountered taxa. Both springs also exhibited elevated arsenic and copper, and a significant difference in iron concentration. Not only do particular temperature, pH, and nutrient levels control the predominance of the cyanobacterial community in hot water springs [10], but also the presence of minor dissolved elements and toxic metals may represent a selective pressure on the inhabiting microbial communities [1,67]. Establishing possible correct correlations between each physicochemical parameter and cyanobacterial diversity could be interesting and challenging, and would require meaningful multivariate statistical analyses [1].

The thermophile *Fischerella* strains are attractive for harboring thermoresistant photosensors (Phytochrome-class) [11,48]. Moreover, they were effective at the production of nitrogenase reductase for nitrogen fixation [68], and removing nutrients (nitrogen and phosphorus) from thermal effluents of a nuclear reactor [11]. The enzymatic activities of *Leptolyngbya* have aroused great interest due to its extensive ability to metabolize pesticides such as glyphosate [69]. In addition, Leptolyngbya within geothermal microalgal consortia are able to increase biomass and neutral lipid production [70]. A strain of *O. sancta* (species most similar to *T. amphibia*) was demonstrated to be an efficient producer of biodiesel at elevated temperatures [60]. Thermophile *Synechococcus* strains have been used in CO_2_ assimilation [71], lipid and biodiesel production [72,73], dye bioaccumulation [74], and poly-β-hydroxybutyrate production [75,76,77]. Therefore, the potential of our strains will need to be investigated in future work.

In the current study, five strains from El Salado and six strains from Papallacta were isolated and characterized. In seven of them, the morphological and molecular analysis complemented each other. Even though we determined the taxonomy of all eleven isolates, further investigation is necessary for strains B3A, P12A and P13A. Ultrastructure data, multiple-locus sequence typing and whole genome sequencing may provide additional information for a more precise identification [2,16]. Both hot springs harbor cosmopolitan cyanobacteria belonging to seven taxa, being *T. amphibia*, *P. raciborskii*, *Nostoc* sp., *F. muscicola*, *K. jovis*, *Synechococcus*, and *Leptolyngbya*. We thus presume that the rest of the isolates are endemic.

This study also contributes to the exploitation of the biotechnological potential of cyanobacterial isolates from geothermal springs, leveraging their inherent resistance to extreme environmental conditions. The strains isolated in the present work are currently being used by the Centro de Nanociencia y Nanotecnología (CENCINAT) in various applied research projects. Specifically, the strain P14A, identified here as *Fischerella muscicola*, has served as a biological component in a hybrid organometallic cell designed for bioelectric energy generation [78]. Moreover, this strain has been employed as a cellular model to assess the toxicity, biocompatibility, and environmental safety of green-synthesized gold and silver nanoparticles [79]. In addition, an ongoing investigation aims to develop hybrid cyanobacteria integrated with metallic nanoparticles to evaluate their genetic and toxicological effects, as well as their interactions with edible plants, to determine both their biotechnological potential and environmental safety (unpublished data). Importantly, the morphological and molecular identification of cyanobacterial isolates achieved in the present study has been essential for enabling and advancing these interdisciplinary applications.

## Figures and Tables

**Table 1:** Physicochemical parameters of the water from the El Salado and Papallacta springs.

Parameters	El Salado	Papallacta
Temperature (°C)	45–48	50–54
pH	6.3–6.6	7.0–7.5
Electrical conductivity (μS/cm)	6850	2080
Cl^−^ (mg/L)	898	404
SO_4_^−2^ (mg/L)	>700	38.4
Na^+^ (mg/L)	>10	138.6
K^+^ (mg/L)	>100	11.4
Ca^++^ (mg/L)	>50	158.4
Mg^++^ (mg/L)	>12	1.9
Arsenic (mg/L)	0.2440	0.686
Copper (mg/L)	<0.05	<0.020
Iron (mg/L)	8	<0.050
Manganese (mg/L)	0.5	0.017
Total alkalinity (mg/L)	>1000	71
Settleable solids (mL/L)	<0.1	<5.0
Total solids (mg/L)	>2000	1522

**Table 2:** Width and length determinations of vegetative cells and heterocysts. The ranges are provided in the first row while below them are the means ± standard deviations. *N* is the sample size.

Strain Code/	Identification	Vegetative Cells			Heterocyst		
Location		N	Width (µm)	Length (µm)	N	Width (µm)	Length (µm)
B2A El Salado	* Tenebriella * *amphibia*	30	14.2–19.4	0.1–4.0		–	–
			(16.4 ± 1.5)	(1.5 ± 1.1)			
B3A El Salado	*Calothrix* sp.	30	1.3–7.9	2.7–11.5	15	3.3–9.5	1.7–9.4
			(5.2 ± 2)	(7.0 ± 2.7)		(5.1 ± 1.7)	(3.8 ± 2.4)
B4A ^a^ El Salado	*Planktothricoides* sp. ^a^	30	2.4–5.9	–		–	–
			(4.4 ± 0.7)				
B5A El Salado	*Leptolyngbya* sp.	30	1.4–3.8	1.5–3.5		–	–
			(2.5 ± 0.6)	(2.5 ± 0.6)			
B6A El Salado	*Synechococcus* sp.	15	3.1–4.3	5.2–10.2		–	–
			(3.8 ± 0.4)	(6.7 ± 1.4)			
P11A Papallacta	*Nostoc* sp.	30	3.9–5.8	2.8–7.8	15	6.5–9.1	8.0–13.0
			(4.7 ± 0.6)	(5.8 ± 1.5)		(7.6 ± 0.7)	(10.5 ± 1.6)
P12A Papallacta	Chroococcales	15	9.8–16.4	9.9–17.1		–	–
			(12.3 ± 2.3)	(12.8 ± 2.2)			
P13A Papallacta	Nostocales	30	3.2–5.9	2.7–6.0	15	2.1–4.0	3.0–4.5
			(4.4 ± 0.8)	(3.9 ± 0.8)		(3.4 ± 0.6)	(3.9 ± 0.5)
P14A Papallacta	*Fischerella* sp.	30	2.7–5.8	3.5–8.1	7	3.8–5.1	4.5–6.8
			(4.3 ± 0.8)	(5.2 ± 1.2)		(4.5 ± 0.4)	(5.2 ± 0.7)
P15A Papallacta	*Leptolyngbya* sp.	30	1.5–3.0	1.0–3.3		–	–
			(2.3 ± 0.3)	(2.5 ± 0.6)			
P38A Papallacta	* Komvophoron jovis *	30	1.6–4.7	1.8–4.3		–	–
			(2.7 ± 0.9)	(3.2 ± 0.6)			

^a^ For vegetative cells of B4A morphotype, the length cannot be measured as their cells lacked of visibility below the microscope.

**Table 3:** Identification of cyanobacteria isolates from two geothermal springs of Ecuador based on morphology and *16S* rRNA and *23S* rRNA gene sequences.

		Molecular Identification				
		*16S* rRNA		*23S* rRNA		
Strain Code	Morphological Identification	Access. No. (Fragment Length bp)	Highest Blast Match (Access. No.) (Identity %)	Access. No. (Fragment Length bp)	Highest Blast Match (Access. No.) (Identity %)	Taxonomic Assignment
B2A	* Tenebriella * *amphibia*	MH090926 (631)	*Tenebriella amphibia* RMCB18 (MT756311) (99.8)	MH101455 (332)	*Trichodesmium erythraeum* IMS101 (NR076432) (92.5)	* Tenebriella amphibia *
B3A	*Calothrix* sp.	MH090927 (630)	*Calothrix* sp. CHAB TP201528 (MT488122) (97.8)	MH101456 (377)	*Cylindrospermopsis raciborskii* NIES-1262 (LC455652) (96.8)	*Calothrix* sp.
B4A	*Planktothricoides* sp.	MH090928 (629)	*Planktothricoides raciborskii* PMC 877.14 (MT984287) (99.8)	MH101457 (390)	*Limnospira fusiformis* SAG 85.79 (CP051185) (92.3)	*Planktothricoides* raciborskii
P11A	*Nostoc* sp.	MH090929 (619)	*Nostoc* sp. PAN-549 (KF921498) (99.2)	MH101458 (379)	*Nostoc* sp. NIES-4103 (AP018288) (96.3)	*Nostoc* sp.
P12A	Chroococcales	MH090931 (632)	*Chlorogloeopsis fritschii* PCC 6912 (MK953013) (95.9)	MH101459 (379)	*Fischerella muscicola* SAG 2027 (AM709634) (94.5)	Chroococcalean cyanobacterium
P13A	Nostocales	MH090930 (633)	*Dendronalium phyllosphericum* CENA369 (NR172569) (98.2)	MH101460 (393)	*Halotia branconii* CENA392 (CP124543) (96.7)	Nostocacean cyanobacterium
P14A	*Fischerella* sp.	MH090932 (622)	*Fischerella muscicola* NDUPC001 (JX876898) (100)	MH101461 (378)	*Fischerella muscicola* SAG 2027 (AM709634) (99.5)	*Fischerella muscicola*

## Data Availability

The original contributions presented in this study are included in the article. Further inquiries can be directed to the corresponding author.
